# Supplementary material for: Preparation and Characteristics of Polyethylene Oxide/Curdlan Nanofiber Films by Electrospinning for Biomedical Applications
Source: Materials (Basel). 2023 May 20;16(10):3863. doi: 10.3390/ma16103863 (PMC10220793; doi:10.3390/ma16103863)
Supplement: Supplementary file 1 [file materials-16-03863-s001.zip › materials-2351816-supplementary.pdf]

Preparation and Characteristics of Polyethylene Oxide/Curdlan Nanofiber Films by Electrospinning for Biomedical Applications

Shu-Hung Lin [1], Sin-Liang Ou [2,*], Hung-Ming Hsu [3] and Jane-Yii Wu [3,4,5,*]

[1] PhD Program of Biotechnology and Industry, College of Biotechnology and Bioresources, Da-Yeh University, Changhua 515, Taiwan; shuhunglin1969@gmail.com
[2] Department of Biomedical Engineering, Da-Yeh University, Changhua 515, Taiwan
[3] Department of Medicinal Botanicals and Foods on Health Applications, Da-Yeh University, Changhua 515, Taiwan; a0933571498@gmail.com
[4] Biotechnology Research and Development Center, Da-Yeh University, Changhua 515, Taiwan
[5] Innovation Incubation Center, Da-Yeh University, Changhua 515, Taiwan
* Correspondence: slo@mail.dyu.edu.tw (S.-L.O.); jywu@mail.dyu.edu.tw (J.-Y.W.)

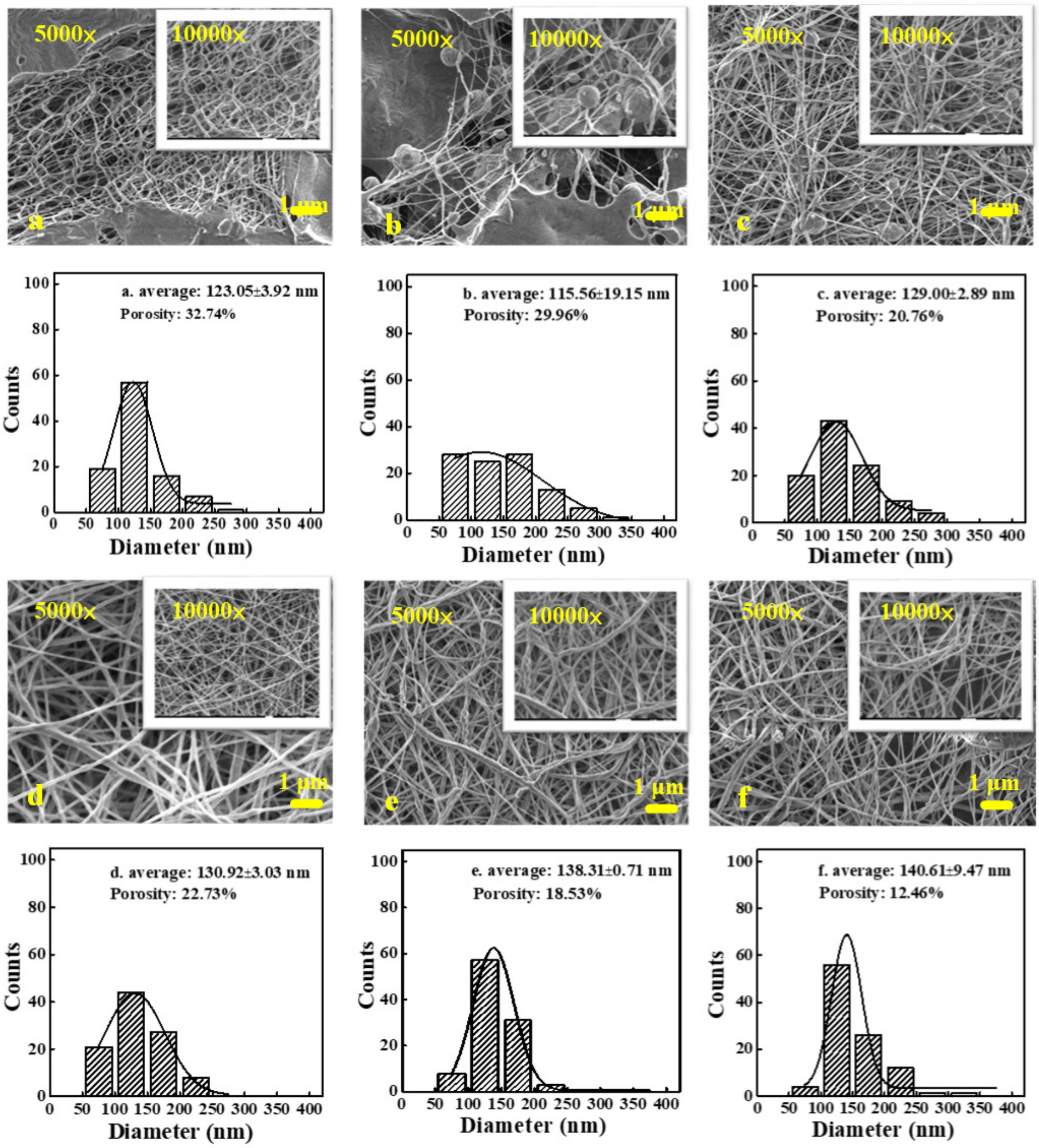

Figure S1. SEM images and fiber-diameter scatter diagrams of PEO/curdlan nanofiber films prepared by electrospinning under various operating voltages of (a) 12, (b) 14, (c) 17.5, (d) 19, (e) 22 and (f) 24 kV. The polymer solution used in the electrospinning process was mixed with 6.0 wt.% PEO and 1.0 wt.% cardlan gum.

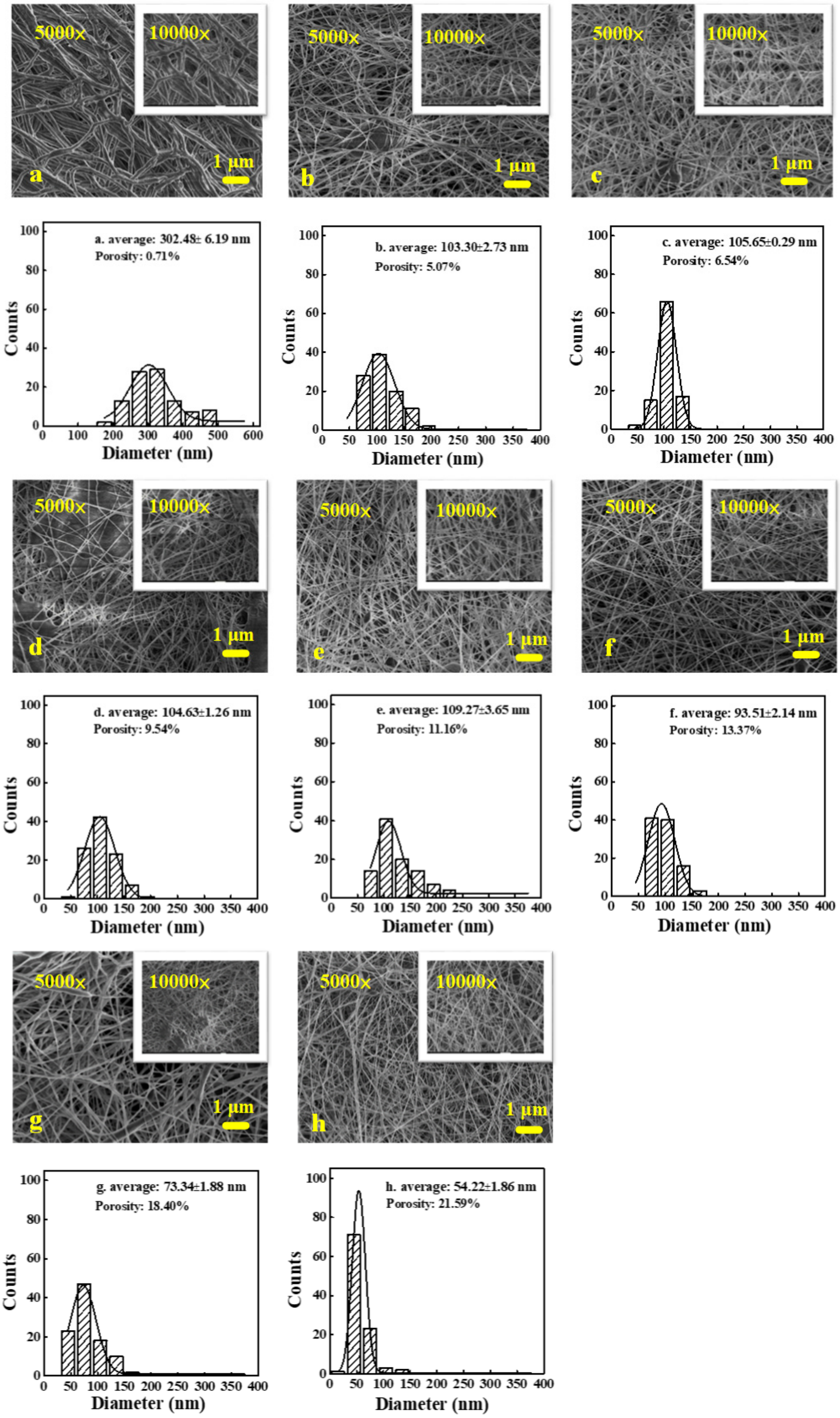

Figure S2. SEM images and fiber-diameter scatter diagrams of PEO/curdlan nanofiber films prepared by electrospinning under various operating voltages of (a) 12, (b) 14, (c) 16, (d) 17, (e) 17.5, (f) 19, (g) 22 and (h) 24 kV. The polymer solution used in the electrospinning process was mixed with 6.0 wt.% PEO and 2.0 wt.% cardlan gum.

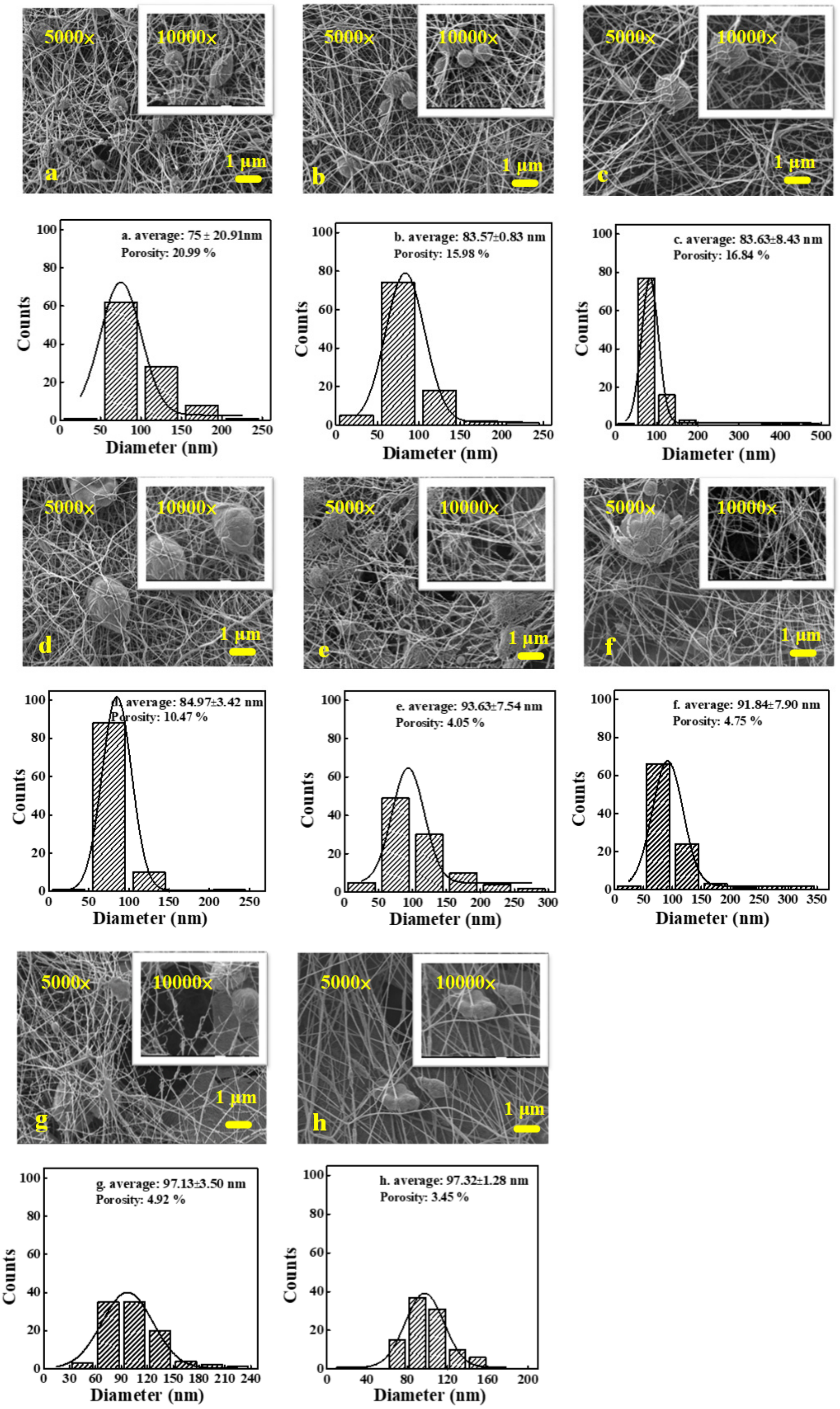

Figure S3. SEM images and fiber-diameter scatter diagrams of PEO/curdlan nanofiber films prepared by electrospinning under various operating voltages of (a) 12, (b) 14, (c) 16, (d) 17, (e) 17.5, (f) 19, (g) 22 and (h) 24 kV. The polymer solution used in the electrospinning process was mixed with 6.0 wt.% PEO and 5.0 wt.% cardlan gum.

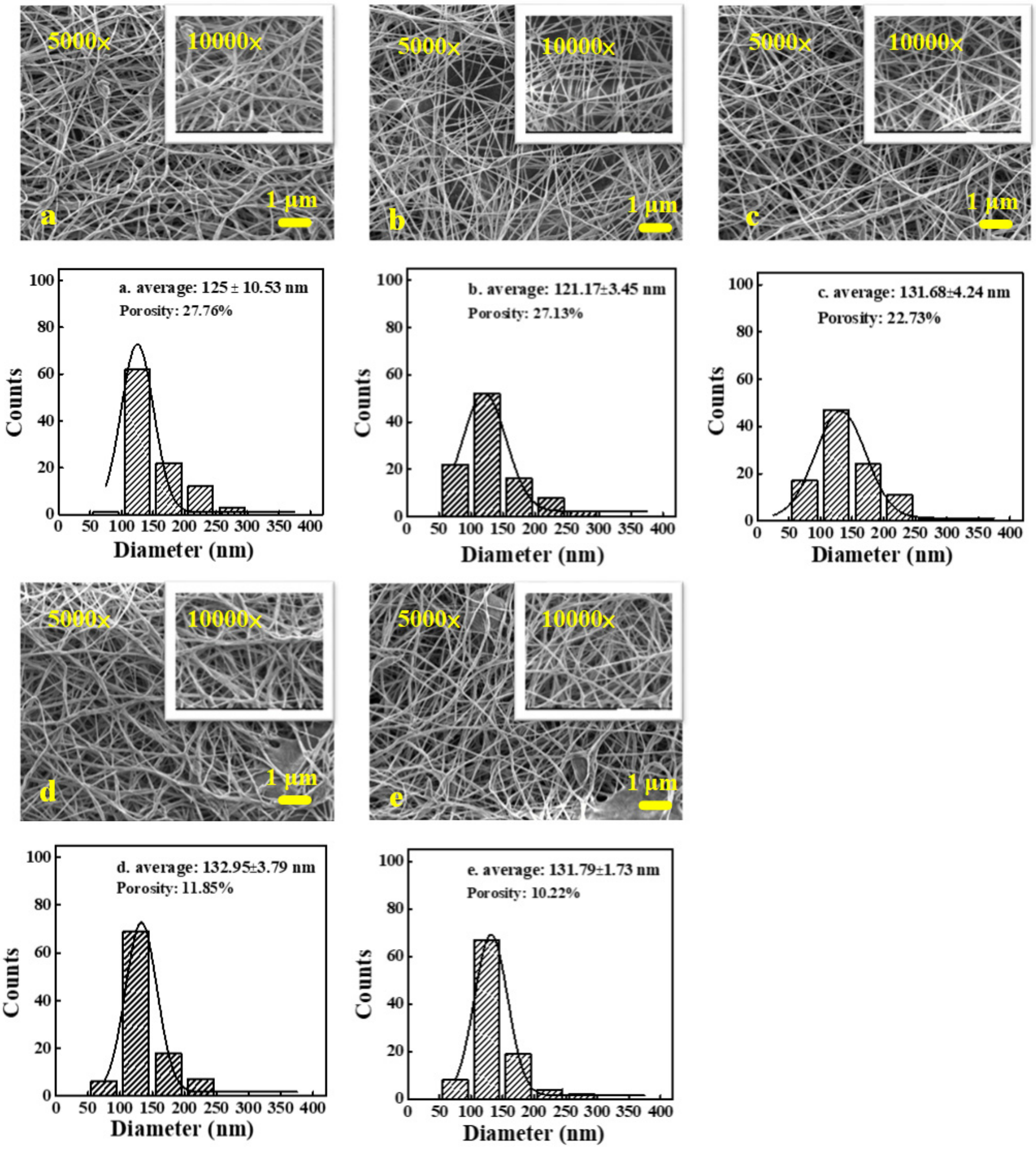

Figure S4. SEM images and fiber-diameter scatter diagrams of PEO/curdlan nanofiber films prepared by electrospinning at various working distances of (a) 12, (b) 13, (c) 15, (d) 17 and (e) 20 cm. The polymer solution used in the electrospinning process was mixed with 6.0 wt.% PEO and 1.0 wt.% cardlan gum.

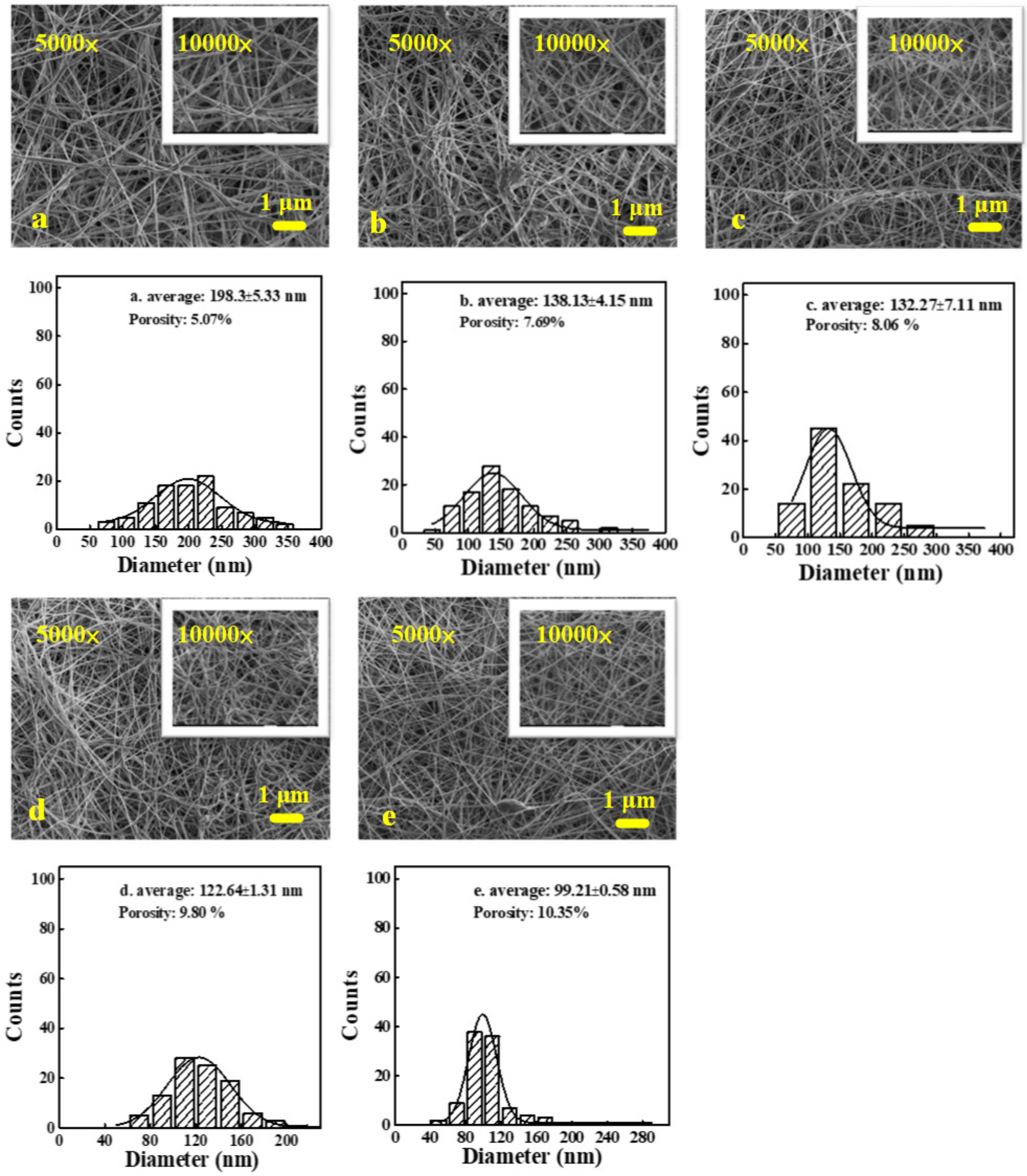

Figure S5. SEM images and fiber-diameter scatter diagrams of PEO/curdlan nanofiber films prepared by electrospinning at various working distances of (a) 12, (b) 13, (c) 15, (d) 17 and (e) 20 cm. The polymer solution used in the electrospinning process was mixed with 6.0 wt.% PEO and 2.0 wt.% cardlan gum.

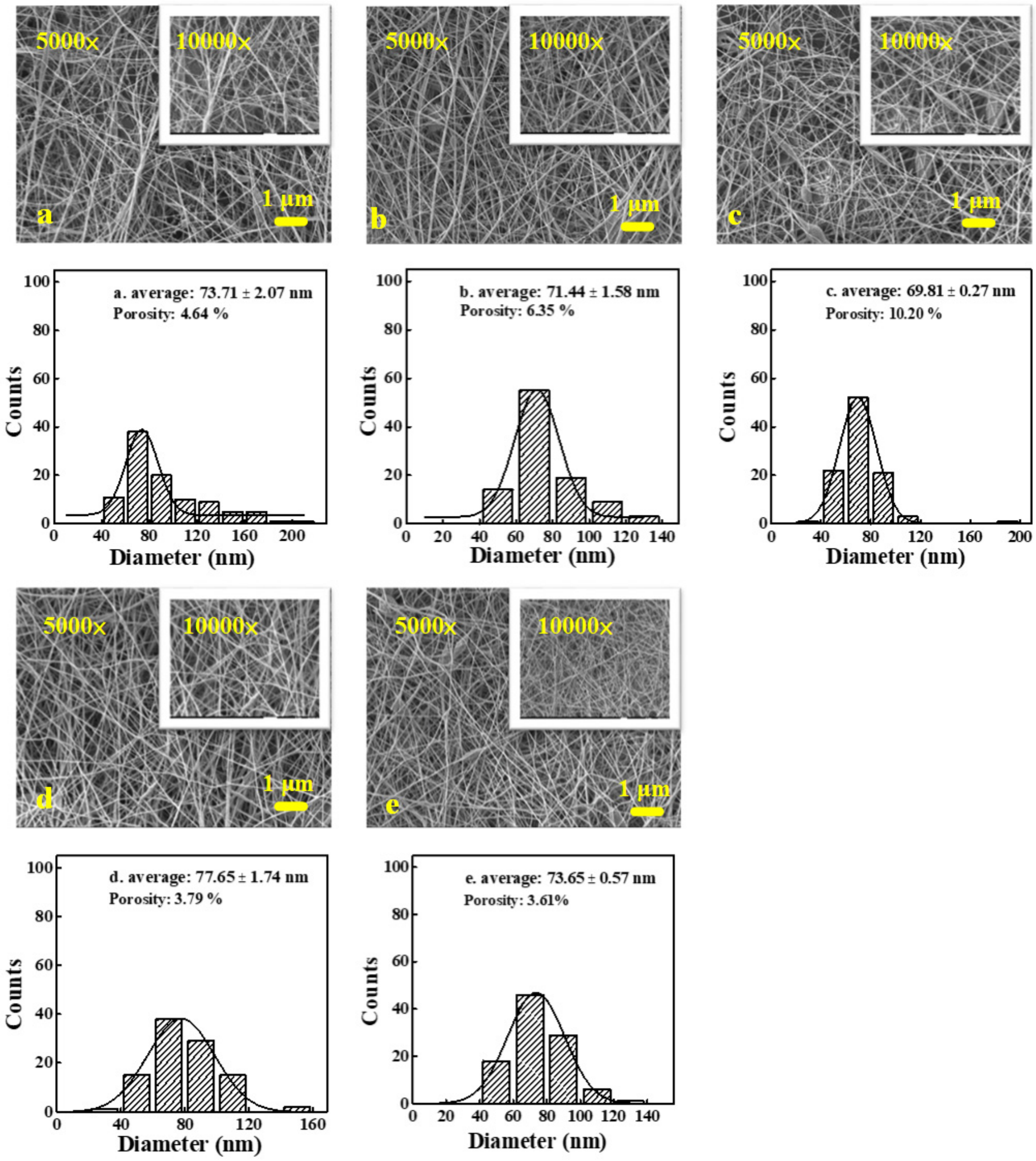

Figure S6. SEM images and fiber-diameter scatter diagrams of PEO/curdlan nanofiber films prepared by electrospinning at various working distances of (a) 12, (b) 13, (c) 15, (d) 17 and (e) 20 cm. The polymer solution used in the electrospinning process was mixed with 6.0 wt.% PEO and 5.0 wt.% cardlan gum.

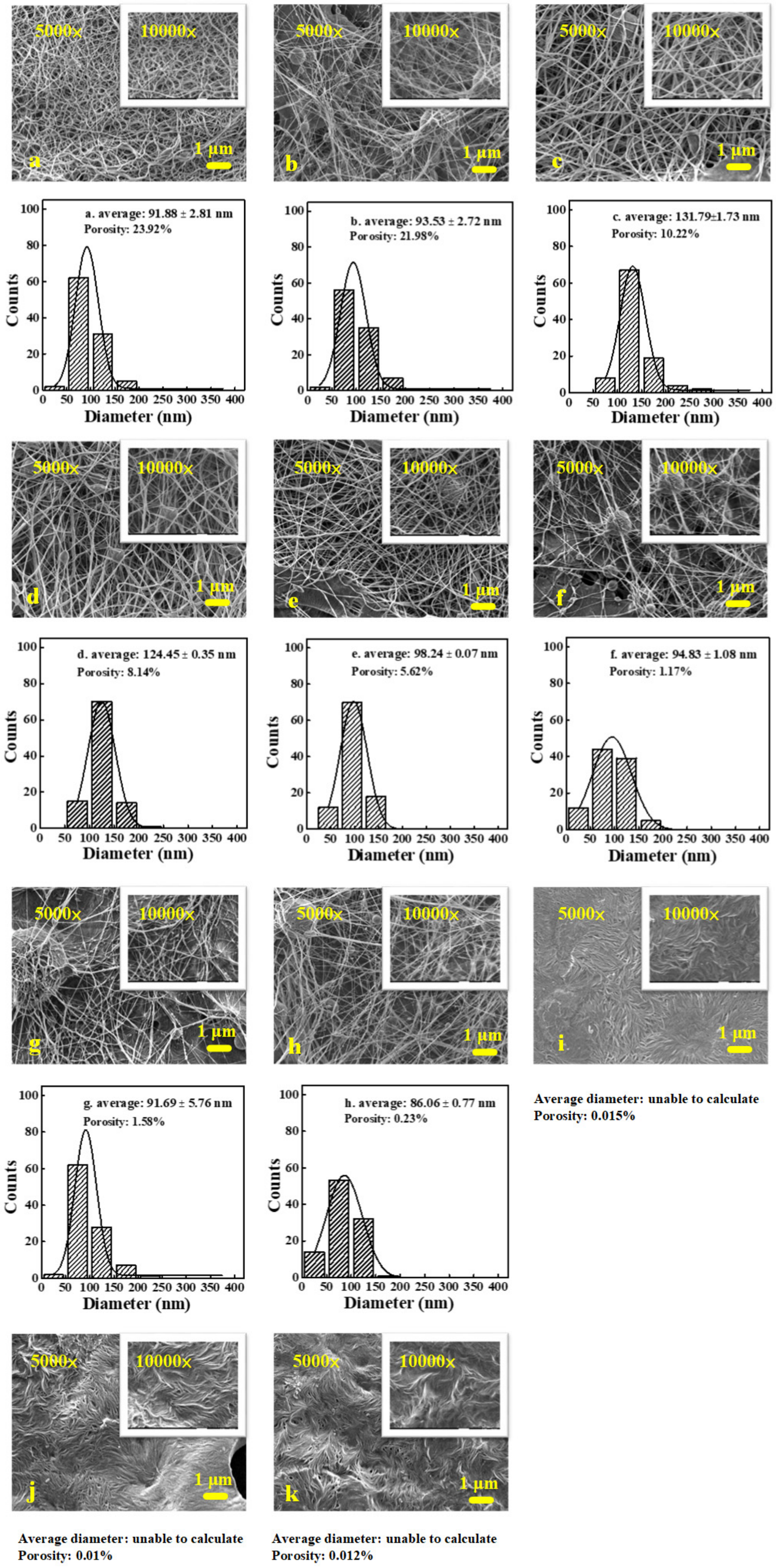

Figure S7. SEM images and fiber-diameter scatter diagrams of PEO/curdlan nanofiber films prepared by electrospinning at various feeding rates of (a) 5, (b) 8, (c) 9, (d) 11, (e) 15, (f) 20, (g) 25, (h) 30, (i) 35 (j) 40 and (k) 50 μL/min. The polymer solution used in the electrospinning process was mixed with 6.0 wt.% PEO and 1.0 wt.% cardlan gum.

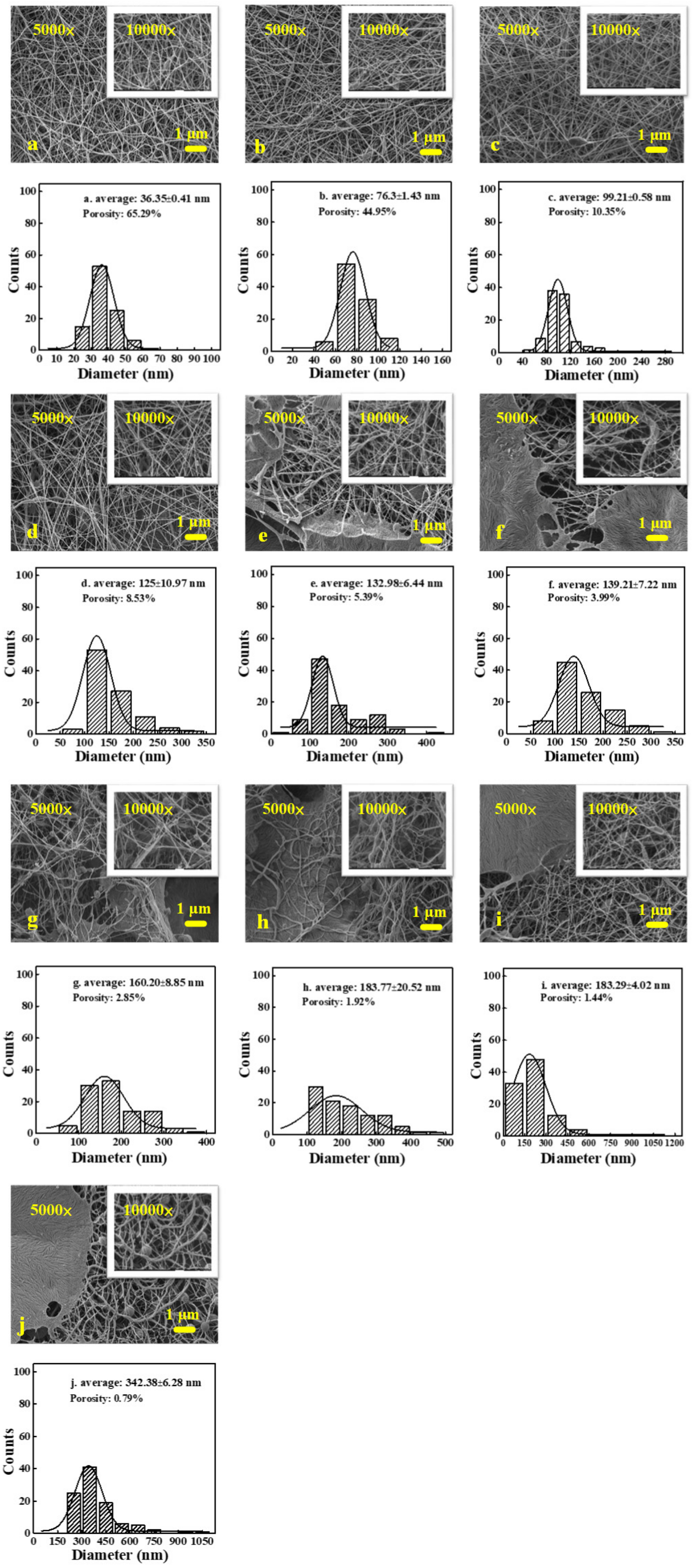

Figure S8. SEM images and fiber-diameter scatter diagrams of PEO/curdlan nanofiber films prepared by electrospinning at various feeding rates of (a) 5, (b) 8, (c) 9, (d) 11, (e) 15, (f) 20, (g) 25, (h) 30, (i) 35 and (j) 40 μL/min. The polymer solution used in the electrospinning process was mixed with 6.0 wt.% PEO and 2.0 wt.% cardlan gum.

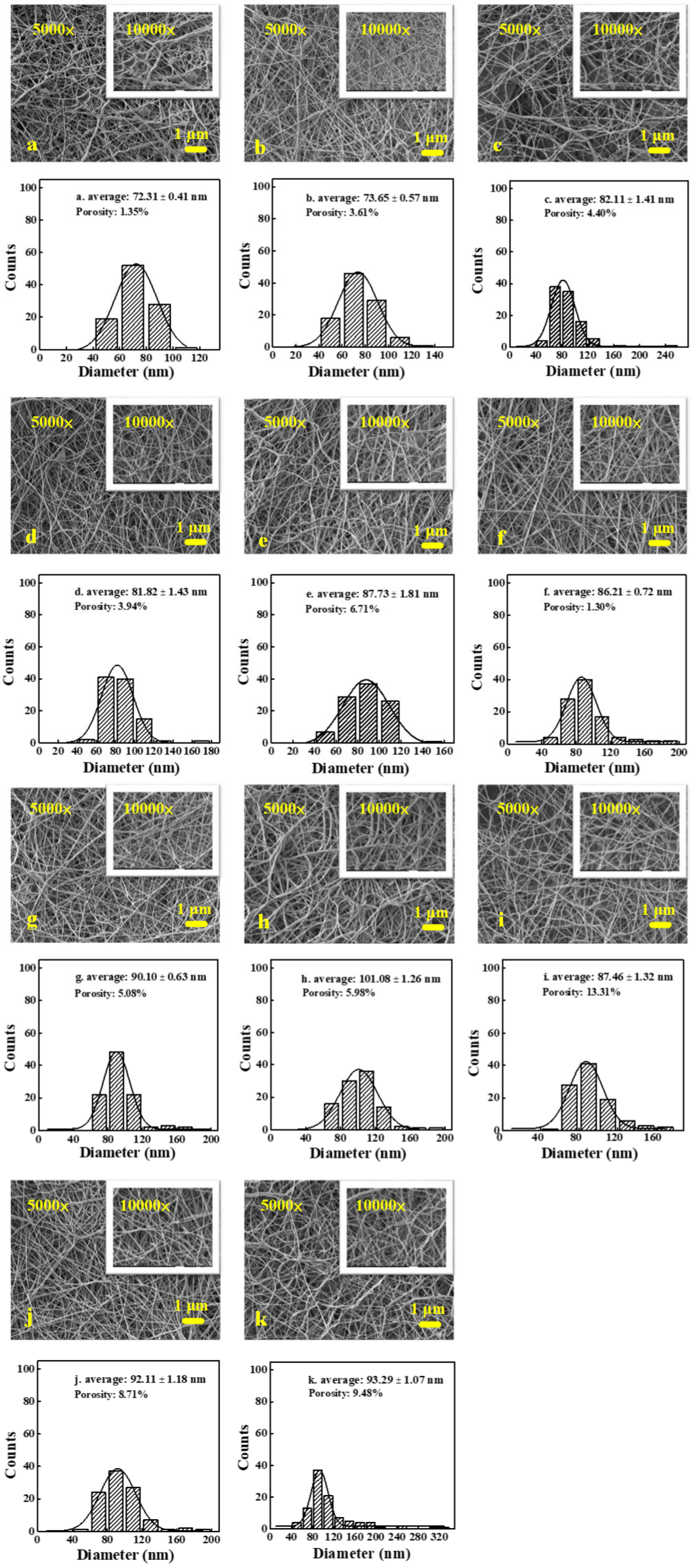

Figure S9. SEM images and fiber-diameter scatter diagrams of PEO/curdlan nanofiber films prepared by electrospinning at various feeding rates of (a) 5, (b) 9, (c) 10, (d) 15, (e) 20, (f) 25, (g) 30, (h) 35, (i) 40, (j) 45 and (k) 50 μL/min. The polymer solution used in the electrospinning process was mixed with 6.0 wt.% PEO and 5.0 wt.% cardlan gum.
